# Supplementary material for: End Sequence Analysis Toolkit (ESAT) expands the extractable information from single-cell RNA-seq data
Source: Genome Res. 2016 Oct;26(10):1397–410. doi: 10.1101/gr.207902.116 (PMC5052061; doi:10.1101/gr.207902.116)
Supplement: Supplemental Material [file supp_gr.207902.116_Supp_Materials.tar.gz › ESAT-esat_v0.1/doc/html/classbroad_1_1core_1_1math_1_1_compute_f_d_r.html]

ESAT: broad.core.math.ComputeFDR Class Reference


ESAT


- Main Page
- Related Pages
- Packages
- Classes
- Files

- Class List
- Class Index
- Class Hierarchy
- Class Members

All Classes Namespaces Files Functions Variables Enumerator Pages

- broad
- core
- math
- ComputeFDR

Static Public Member Functions |
List of all members

broad.core.math.ComputeFDR Class Reference

|  |  |
| --- | --- |
| Static Public Member Functions | |
| static double | FDR (EmpiricalDistribution observed, EmpiricalDistribution[] random, double k) |
|  | |
| static double | FDR (Map< String, Double > observed, EmpiricalDistribution[] random, double k) |
|  | |
| static double | FDRExact (Map observed, Map[] expected, double k) |
|  | |
| static double | FDRExactPrint (Map observed, Map[] expected, double k) |
|  | |

## Member Function Documentation

|  |  |  |  |
| --- | --- | --- | --- |
| static double broad.core.math.ComputeFDR.FDR | ( | EmpiricalDistribution | *observed*, |
|  |  | EmpiricalDistribution[] | *random*, |
|  |  | double | *k* |
|  | ) |  |  |

static

|  |  |  |  |
| --- | --- | --- | --- |
| static double broad.core.math.ComputeFDR.FDR | ( | Map< String, Double > | *observed*, |
|  |  | EmpiricalDistribution[] | *random*, |
|  |  | double | *k* |
|  | ) |  |  |

static

|  |  |  |  |
| --- | --- | --- | --- |
| static double broad.core.math.ComputeFDR.FDRExact | ( | Map | *observed*, |
|  |  | Map[] | *expected*, |
|  |  | double | *k* |
|  | ) |  |  |

static

|  |  |  |  |
| --- | --- | --- | --- |
| static double broad.core.math.ComputeFDR.FDRExactPrint | ( | Map | *observed*, |
|  |  | Map[] | *expected*, |
|  |  | double | *k* |
|  | ) |  |  |

static

---

The documentation for this class was generated from the following file:

- java/broad/core/math/ComputeFDR.java


---

Generated on Fri Aug 15 2014 15:10:52 for ESAT by  

 1.8.7
